# Recovery of Phenolic Compounds from Red Grape Pomace Extract through Nanofiltration Membranes

**DOI:** 10.3390/foods9111649

**Published:** 2020-11-12

**Authors:** Jaime A. Arboleda Mejia, Arianna Ricci, Ana S. Figueiredo, Andrea Versari, Alfredo Cassano, Giuseppina P. Parpinello, Maria N. De Pinho

**Affiliations:** 1 Department of Agricultural and Food Sciences, University of Bologna, Piazza Goidanich 60, 47521 Cesena (FC), Italy; jaime.arboleda@studio.unibo.it (J.A.A.M.); arianna.ricci4@unibo.it (A.R.); andrea.versari@unibo.it (A.V.); 2 Interdepartmental Centre for Industrial Agrofood Research, University of Bologna, Via Quinto Bucci 336, 47521 Cesena (FC), Italy; 3 CeFEMA/Departmental Area of Chemical Engineering, Instituto Superior de Engenharia de Lisboa, Instituto Politécnico de Lisboa, Rua Conselheiro Emídio Navarro 1, 1959-007 Lisbon, Portugal; asofia@deq.isel.ipl.pt; 4 CeFEMA/Department of Chemical Engineering, Instituto Superior Técnico, Universidade de Lisboa, Av. Rovisco Pais 1, 1049-001 Lisbon, Portugal; marianpinho@tecnico.ulisboa.pt; 5 Institute on Membrane Technology, ITM-CNR, c/o University of Calabria, via P. Bucci, 17/C, I-87036 Rende (Cosenza), Italy; a.cassano@itm.cnr.it

**Keywords:** antioxidants, biorefinery, process optimization, fractionation, sustainability, winemaking exploitation

## Abstract

The winemaking process generates a large amount of residues such as vine shots, stalks, grape pomace, and wine lees, which were only recently considered for exploitation of their valuable compounds. The purpose of this work was to investigate the performance of nanofiltration for the recovery of phenolic compounds, with bioactive capacity like antioxidant, from red grape pomace extract. Four membranes were compared in this study—three cellulose acetate (CA series: lab-prepared by phase inversion) and one commercial (NF90). All membranes were characterized for their hydraulic permeability and rejection coefficients to reference solutes like saccharose, glucose, raffinose, polyethylene glycol, sodium chloride, and sodium sulfate. Permeation flowrates and rejection coefficients towards total phenolics content, antioxidant activity, proanthocyanidins, glucose and fructose were measured in the nanofiltration of grape pomace extract using selected operating conditions. Among the investigated membranes, the CA400-22 exhibited the highest permeate flux (50.58 L/m^2^ h at 20 bar and 25 °C), low fouling index (of about 23%), the lowest rejection coefficients towards the reference solutes and the best performance in terms of separation between sugars and phenolic compounds. Indeed, the observed rejections for glucose and fructose were 19% and 12%, respectively. On the other hand, total phenolics content and proanthocyanidins were rejected for 73% and 92%, respectively.

## 1. Introduction

The wine industry, with estimated worldwide annual grape processing of 78 million tons, produces one of the most abundant agro-waste resources represented by leaves, stems, pomaces (including grape skins and seeds) and lees [1].

The oenological wastes are usually conferred to distilleries for ethanol and tartaric acid recovery, or alternatively used as animal feed, fertilizers, or vineyard amendment [2,3]. However, organic waste disposal practices might have environmental impacts, causing soil and groundwater contamination, unpleasant odors, plant pathogen vectors (pests and flies) attraction, and the depletion of oxygen in the soil and groundwater, caused by tannins and other compounds degradation [4,5]. In recent times, the valorization of agro-wastes was boosted through the biorefinery approach, offering a great potential for wine companies to reduce the environmental impact and create profit, adopting sustainable practices that deal with the principles of the Circular Economy [6].

Among wastes, grape pomaces constitute a valuable source of polyphenolic compounds (flavonoids, tannins, and benzoic acids derivatives), which are biomolecules of both technological and nutraceutical interest, acting as antioxidants in biological matrices, through the inhibition of lipid peroxide radicals and reactive oxygen species [7,8,9]. In addition, the pomaces contain molecules of biological interest, such as essential oils, proteins, minerals, sugars, dietary fibers, and pectins, which might enhance the nutritional value of food ingredients and bioactive supplements obtained by oenological waste valorization [4,10,11]. Several studies demonstrated the protection activity of grape seed and pomace extracts against microbial inflammation, cancer, and degenerative diseases [12,13,14,15]. A virtuous oenological chain might consider the valorization of massive pomace wastes by adopting highly sustainable processes for the recovery of polyphenols. In this view, several research studies were carried out for substituting conventional solvent extraction procedures and avoiding impact swapping [16,17]. Among them, ultrasound and microwaves-assisted extractions in combination with aqueous solvent as well as non-conventional (solvents free) methods showed their potential in guaranteeing safe and high-quality of polyphenolic extracts [18,19,20,21]. Caldas et al. [22] found that ultrasound-assisted extraction showed the best phenolic recovery from grape skin of sparkling production, in comparison to conventional methods and microwave-assisted extraction. High amounts of anthocyanins and tannins were recovered from fermented grape pomace through a subcritical water extraction method [23]. The use of β-cyclodextrin was also proposed as a green extraction technique for isolating bioactive phenolics from grape pomace [24]. Recently, Meini et al. [25] reported the use of grape pomace as carbon source and inducer in submerged fermentation cultures of *Aspergillus niger*, proposing new routes for grape pomace valorization.

In this context, membrane processes represent a new frontier in bioactive compound exploitation being a powerful alternative to conventional methodologies, due to their low energy and no-additives requirements, mild temperature and pressure conditions, high separation efficiency and easy scale up [17]. In particular, pressure-driven membrane operations such as microfiltration (MF), ultrafiltration (UF), nanofiltration (NF), and reverse osmosis (RO) were largely investigated in the last few years, for the separation, purification, and concentration of natural antioxidants from several agro-food by-products, including olive mill wastewaters [26], artichoke brines [27], bergamot juice [28], and wine lees [29]. The use of UF membranes with molecular weight cut-off (MWCO) in the range 2–100 kDa was recently investigated for the fractionation of phenolic compounds from subcritical water grape pomace extract and the separation of these compounds from other co-extracted components [30]. On the other hand, the application of NF for the recovery of these compounds from grape pomace is still a matter of research.

In this work, we evaluated the feasibility of phenolic compounds recovery from red grape pomace through a combination of ultrasound-assisted enzymatic extraction, followed by a membrane separation process. The extraction parameters for grape pomace, were optimized by considering different enzymatic dosages and the number of ultrasound cycles. Afterwards, the extract was fractionated by means of three flat-sheet cellulose acetate membranes prepared in the laboratory. Membranes were characterized for their hydraulic permeability and rejection towards reference solutes. Their performances in terms of productivity, fouling index, cleaning efficiency, and selectivity towards target compounds was evaluated and compared with that of a commercial NF membrane. The grape pomace extract and the membrane fractions were characterized in terms of total phenolics content, proanthocyanidins, turbidity, antioxidant activity, glucose, and fructose. Results were discussed in terms of composition of the extracts and membrane performances to improve their application for bioactive compound extraction from food waste.

## 2. Materials and Methods

### 2.1. Red Grape Pomace

The red grape pomace (a mixture of 60% cabernet sauvignon, 30% Sangiovese, and 10% Syrah) was collected from a local winery (Terre Naldi, Faenza, Italy), stored at −20 °C and dried at 50 °C for 24 h, before use.

### 2.2. Ultrasonic-Assisted Enzymatic Extraction

The extraction of polyphenols from grape pomace was carried out by means of an enzymatic hydrolysis with pectolytic enzymes (Lafase^®^ He Grand Cru from Laffort, Bordeaux, France) coupled with ultrasonic irradiation, according to the methodology reported by [31], with some modifications related to the sonication power and number of operation cycles. In particular, Ultrasonic irradiation was applied by means of a High Intensity Ultrasonic Liquid Processor (VCX 50s-VC 75, Sonics, Newtown, CT, USA), at 16 W, for a different number of cycles (from 4 to 10), for 1 min.

To optimize the conditions a three-level Design of Experiment (DoE was used in this study. In brief, 0.250 g of dried grape pomace were mixed with 20 mL of succinic acid (50 mM)—borax (50 mM) buffer (pH 4.0) at 40 °C for 2 h exposure time (t_(h)_) and three enzymatic dosages (10, 20 or 30 mg g**^−^**^1^ of dried pomace). The DoE consisted of thirteen trial replicates. Optimal conditions were settled according to the results of DoE and used to obtain a 5 L extract to feed the nanofiltration system.

Before the NF treatment, the solution was prefiltered by using a nylon filter with a hole diameter of 3 mm, in order to achieve a solid–liquid separation.

### 2.3. Nanofiltration Membranes

Three cellulose acetate (CA) laboratory-made membranes coded as CA400-22, CA316-70, and CA316 were prepared at the Department of Chemical Engineering of the Instituto Superior Tecnico (Portugal), according to the phase inversion method [32]. Among them, the membrane CA316-70 was further subjected to annealing treatment. Membrane-casting solutions and film-casting conditions are reported in Table 1. Cellulose acetate, acetone, formamide, and magnesium perchlorate were of analytical grade (Merck Millipore, Darmstadt, Germany). In parallel, permeation experiments with a commercially available thin-film composite NF membrane (NF90, Dupont Filmtec, Wilmington, DE, USA) were carried out. Its characteristics are reported in Table 2.

All membranes were characterized for water permeability and rejection coefficients to reference solutes including d-(+)-glucose, raffinose, polyethylene glycol (PEG 1000 Da), sodium chloride, sodium sulfate, and ethanol. Tests were carried out according to a total recycle configuration (recycling both permeate and retentate in the feed reservoir) in selected conditions of temperature (25 ± 1 °C) and feed flowrate (0.8 L/min), by using feed solutions with a solute concentration of 600 ppm. The water permeability of the membranes was obtained by the linear regression of the water flux against the transmembrane pressure (TMP). The average crossflow velocity was 0.17m/s and the corresponding Reynolds number was 4000. A force balance (pressure forces = shear forces) in the feed chamber of the permeation cells allowed the estimation of non-significant shear forces, due to the negligible pressure drop in the feed circulation stream.

### 2.4. Experimental Set-Up and Procedures

**Figure 1:**
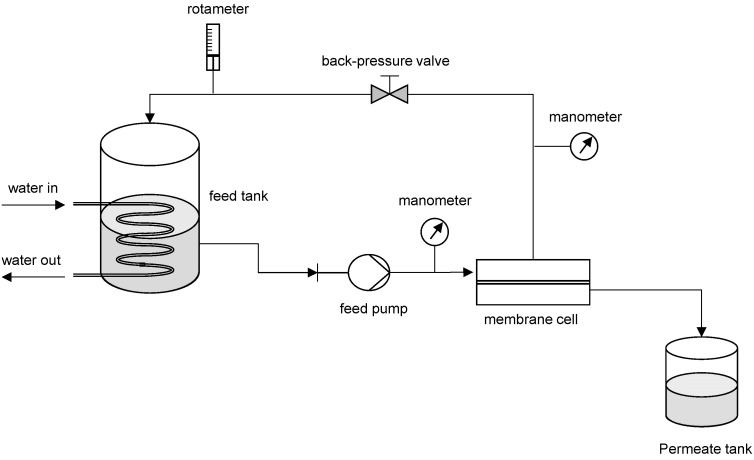
Schematic of lab-scale filtration unit including—water out; water in; feed tank; rotameter; back-pressure valve; manometer; membrane cell; feed pump; and permeate tank.

**Figure 2:**
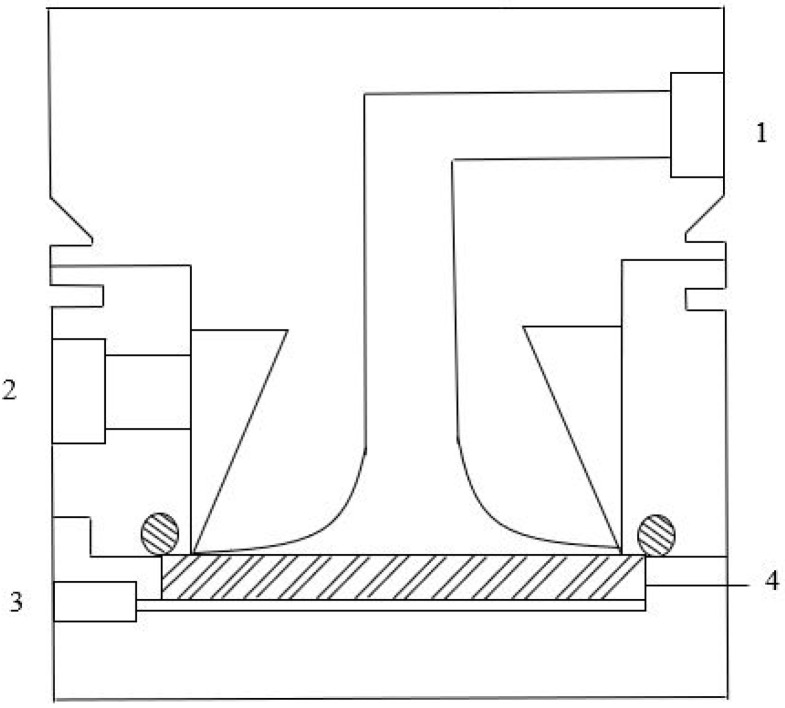
Cross-section of the membrane cell. 1. Feed inlet; 2. Feed outlet; 3. Permeate stream; 4. Porous stainless-steel plate (membrane support). Figure adapted from [34].

The permeation experiments were carried out in the laboratory filtration unit illustrated in Figure 1. The core of the set-up included a flat plate stainless steel cross-flow cell, with two detachable parts separated by a porous plate supporting the membrane with an active filtration area of 13.2 × 10^−4^ m^2^. An important feature of the membrane cell is the conical shape of the upper part—pressurized feed side—to induce a feed turbulent flow and minimize solute accumulation at the membrane surface (Figure 2).

Before the permeation tests, the membranes were compacted by circulation of deionized water at constant operating pressure of 35 bar for 2 h, in order to minimize any effect on the membrane structure during the tests, due to the pressure to which they are subjected, and to obtain a steady flux. Experiments were carried out in recirculation mode with the retentate being recirculated into the feed tank, in selected conditions of temperature (25 ± 1 °C) and pressure (20 bar). Approximately 5 L of pomace extract were used as feed.

The permeate flux (*J_p_*), expressed as L/m^2^ h, was calculated as follows:

(1) $$J_{p}=\frac{V_{p}}{A\times t}$$

where *V_p_* is the volume of permeate collected during the time interval *t* and *A* is the membrane surface area of permeation.

To investigate the causes of the permeate flux reduction compared to the one of water permeation, the water permeate flux before and after the experiments with grape pomace was measured. The fouling index (*FI*) was estimated by comparing the pure water permeability before and after the NF experiments, according to the following equation:

(2) $$FI\left(\%\right)=\left(1-\frac{W_{p1}}{W_{p0}}\right)\times 100$$

where *W_p_*_0_ and *W_p_*_1_ are the pure water permeability, before and after each nanofiltration run, respectively.

To measure the water permeability after the NF process, the grape pomace extract was removed from the plant. Then, the plant was filled with deionized water and the water permeability at a temperature of 25 ± 1 °C at a feed flow rate of 0.8 L/min was measured. Afterwards, the membranes were washed four times by flushing with deionized water at a temperature of 25 ± 1 °C; hence, deionized water was recirculated for 30 min without any pressure in the system at a temperature of 25 ± 1 °C and at a feed flowrate of 0.8 L/min, separately collecting the permeate stream. At the end of the process, the deionized feed water and the permeate collected during this time were discarded. The process was repeated with a new amount of deionized water for 30 min in the same conditions and the permeability with deionized water was re-measured. The cleaning efficiency (*CE*) was evaluated by using the water flux recovery, according to the following equation:

(3) $$CE\left(\%\right)=\left(\frac{W_{p2}}{W_{p0}}\right)\times 100$$

where *W_p_*_2_ is the water permeability measured after the cleaning process.

### 2.5. Analytical Evaluations

Feed (F), permeate (P) and retentate (R) samples from NF experiments were immediately refrigerated and kept at 4 °C until analyzed for total phenolics, proanthocyanidins content, antioxidant capacity, sugars (glucose and fructose), pH, and turbidity.

The total phenolics content (TPC) was determined through the Folin-Ciocalteau method, based on the redox reaction of phenolic compounds with a mixture of phosphotungstic (H_3_PWO_12_O_40_) and phosphomolybdic (H_3_PMO_12_O_40_) acids in an alkaline medium to create a blue complex [35]. The reaction was performed by mixing a 0.250 mL sample aliquot, 0.5 mL of Folin-Coicalteu reagent (diluted 1:4) and 1.75 mL of a 15% sodium carbonate solution. After 1 h, the absorbance was read by using an UV-vis spectrophotometer (UV-1700 Shimadzu), at a wavelength of 725 nm. The results were expressed as mg of Gallic Acid Equivalent (GAE)/100 g dry matter.

The quantification of proanthocyanidins was carried out by means of a spectrophotometric assay with the 4-dimethyl-amino cinnamaldehyde reagent (DMAC), according to the method previously reported [36]. Results were expressed as mg of (+)-Catechin Equivalents (CE)/100 g dry weight. The monosaccharides (d-glucose and d-fructose, expressed as mg/100 g dry matter) were determined by an enzymatic assay (Megazyme, Chicago, IL, USA). The analysis was based on the sequential reactions that begin after the addition of hexokinases (HK) and phosphoglucose isomerase (PGI), for the determination of glucose and fructose, respectively. The amount of NADPH formed throughout the reactions, which was measured spectrophotometrically at an absorbance of 340 nm, was stoichiometric with the amount of d-glucose and d-fructose present in the sample. The pH value was measured by potentiometry (HANNA 209 pH meter, Merk, Darmstadt, Germany), whereas turbidity (expressed as NTU) was measured by means of a compact infrared turbidity meter (AL250T-IR, Aqualytic^®^, Dortmund, Germany).

The rejection of NF (*R*) membranes towards specific compounds was calculated according to the following equation:

(4) $$R\left(\%\right)=\left(1-\frac{C_{p}}{C_{f}}\right)\;\times \;100$$

where *C_p_* and C*_f_* are the concentration of specific compounds in the permeate and feed samples stream, respectively.

The antioxidant capacity was measured by means of the 2,2′-azinobis-(3-ethylbenzothiazoline-6-sulfonic acid (ABTS) assay according to Re et al. [37] at 734 nm, and calculated as percentage of radical scavenged:

(5) $$AC\left(\%\right)=\left(\frac{R_{0-}R_{1}}{R_{0}}\right)\times 100$$

where *R*_0_ is the absorbance value of the blank and *R*_1_ is the absorbance of the sample. Results were reported as percentage of the radical scavenging of the sample. This method was based on the ability of antioxidants to interact with the ABTS radical, decreasing its absorbance to 734 nm. In brief, a radical solution was prepared, which was based on 7 mM ABTS (2,2′-azino-bis-(-3-ethylbenzothiazoline-6-sulfonic acid)) and 2.45 mM sodium persulfate, then, the solution was left in the dark, at room temperature, for 16 h before use. Subsequently, the ABTS stock solution was added in acetate buffer to an absorbance range of 0.68 to 0.70, at a wavelength of 734 nm. For analysis, 2.9 mL of the ABTS reagent solution was mixed with 0.1 mL microliters of the sample and the blank was prepared by adding 2.9 mL ABTS reagent solution to 0.1 mL distilled water. After 300 min, the absorbance was read by using an UV–Vis spectrophotometer at 734 nm. Moreover, the antioxidant capacity AC-DPPH was evaluated using 1,1-diphenyl-2-picryl-hydrazyl free radical (DPPH) [38] characterized by an intense purple red color, which bleaches when reduced in the presence of a molecule capable of neutralizing by transfer of proton or electron. Shortly, the DPPH reagent was dissolved in pure methanol, subsequently it was brought to an absorbance of 0.7 (±0.010). The solution was kept refrigerated at 4 °C, before use. For analysis, 2.9 mL of the DPPH solution was mixed with 0.1 mL of the sample, and the blank was prepared by adding 2.9 mL DPPH solution to 0.1 mL distilled water. Subsequently, the solution was stirred and kept in the dark at room temperature. After 1 h, it was read on a spectrophotometer at a wavelength of 517 nm, against methanol. Both analyses were carried out by using an UV–Vis spectrophotometer (Cary 60, Agilent Technologies, Santa Clara, CA, USA). Results were expressed as percentage of radical scavenging activity.

### 2.6. Statistical Analysis

Data are presented as mean values ± standard deviation (SD) obtained from three replicated analyses. Significant differences were evaluated by means of One-Way ANOVA analysis of variance (*p* ≤ 0.05) and Tukey’s HSD post-hoc test, using statistical software Minitab^®^ 17.10 (Minitab, Ltd., Coventry, UK).

## 3. Results and Discussion

### 3.1. Membrane Characterization

The rejections for the NF membranes towards a set of reference solutes, salts, and ethanol, decreased as follows (Table 3):

NF90 > CA316-70 > CA316 > CA400-22

The NF90 and the CA316-70 membranes presented rejections towards sodium sulfate of 99% and 97%, respectively, and to sodium chloride of 95% and 77%, respectively. On the other hand, the CA400-22 and CA316 membranes presented lower rejections to sodium sulfate (47% and 86%, respectively) and to sodium chloride (10% and 27%, respectively). However, there was still a differentiation between the two salts, as characteristic of NF membranes. The result reflected the characteristic behavior of NF membranes.

The water permeability data (see Figures S1–S4 in supplementary data) were in agreement with typical values of NF membranes, with the CA400-22 membrane exhibiting the highest value (8.34 L/m^2^h bar) and the NF90 membrane showing the lowest one (3.75 L/m^2^ h bar).

### 3.2. Grape Pomace Extract

Phenolic compounds were extracted from grape pomace by means of an optimized ultrasonic-assisted enzymatic treatment as follows. Enzymatic dosage—10 mg g**^−^**^1^; number of ultrasound cycles—4 with 1-min duration for each cycle. The physico-chemical composition of the grape pomace extract is reported in Table 4.

The red grape pomace extract had a pH of 4.0 due to the use of the buffer solution, which contained succinic acid (C_4_H_6_O_4_) and sodium borate (Na_2_B_4_O_7_·10H_2_O). The total phenolics content in the extract resulted on 260 mg GAE/100 g, using a solid–solvent ratio of 1:80 (*w/v*). Similar values were obtained by Nayak et al. [39], which reported a total phenolics content of 427.9 mg GAE/100 g for the water extraction (solid–solvent ratio of 1:20 *w/v*) compared to the higher value of 801.6 mg GAE/100 g obtained by the same author, with water–ethanol extraction (solid–solvent ratio of 1:20 *w*/*v*) of the Cabernet Sauvignon grape pomace. Differences in the extraction yield of polyphenolic compounds from grape pomaces might be discussed in the light of several technological factors, including grape variety and soil management, winemaking conditions of solvent type (aqueous, organic) and extraction technology [40].

The red grape pomace extract contained 46 mg/100 g dw and 403 mg/100 g dw of glucose and fructose, respectively. The glucose content in the grape pomace was low (0.046% dw), with an increase in the fructose extracted (0.40% dw); both sugars were several orders of magnitude lower than previous reports after Pedras et al. [20], which obtained glucose and fructose contents of 2.8% dw and 3.1% dw, respectively, in their red grape pomace extract.

The content of proanthocyanidins in the extract was 49.0 mg CE/100 g dw and higher values (in the range 100–250 mg catechin/100 g) were reported for different grape pomace varieties, including white grape varieties such as Sauvignon Blanc and Chardonnay, and red grape varieties such as Cabernet Sauvignon and Carménère [41]. It is worth noticing that values estimated by these authors include monomeric, oligomeric, and polymeric flavan-3-ols and that the total polyphenols obtained using stepwise methanol/water and acetone/water extractions were 10 order higher than those obtained by enzymatic-assisted extraction in this study [41].

The antioxidant capacity could be considered to be strong, intermediate, or weak when the radical DPPH scavenging activity was above 70%, between 50 and 70%, and below 50%, respectively [42]. According to the cited ranking, the red grape pomace extract had a weak antioxidant activity, as measured by the DPPH radical scavenging assay (16 ± 0.3%), and showed increasing effectiveness when the ABTS was used as the reference radical (41 ± 3.5%). In both cases, the relatively low content of polyphenolic compounds in the extract, represented a limiting factor for their application as antioxidant; this limitation might be overcome by careful selection of the raw materials and optimization of the extraction parameters.

### 3.3. Membrane Productivity

Table 5 reports the average permeate fluxes (*J_p_*) of the grape pomace extract nanofiltration at 20 bar, with the four NF membranes. The CA400-22 membrane showed the highest permeate flux with a value of 50.58 L/m^2^ h, while the CA316 and the CA316-70 membranes showed quite similar permeate flux values (44.44 and 43.38 L/m^2^ h, respectively); on the other hand, the NF90 membrane showed the lowest permeate flux (26.09 L/m^2^ h). These results were in agreement with the rejection values measured for different solutes and data of water permeability. In particular, a strong correlation between the cut-off of the membranes and the permeate flux values could be inferred.

Commercial polymeric membranes such as DL2540 and GE2540 (from Osmonics, Minnetonka, MN, USA) with cut-off of 150–300 and 1000 Da, respectively, produced permeate fluxes of the order of 23–28 L/m^2^ h, when processing aqueous extracts from distilled fermented grape pomace at 8 bar, in the full recycle mode [43]. In this case, the effects observed in terms of flux declining were not in direct relation to the pore size, suggesting that the chemical nature of the membrane material plays a key role in the membrane performance.

### 3.4. Fouling Index and Cleaning Efficiency

Membrane fouling plays a leading role at different stages of the membrane filtration process and in the selection of a membrane for a specific application. Once membrane fouling occurs, it reduces the permeate flux, increases feed pressure, reduces productivity, increases membrane maintenance and operation costs due to membrane cleaning, and decreases the membrane lifetime [44]. The fouling index for the investigated membranes was calculated by measuring the water permeability before and after the filtration of the red grape pomace extract. According to the results in Table 6, the NF90 membrane showed the highest fouling index (40.53%) followed by the CA316-70 membrane (35.97%), the CA400-22 membrane (23.38%), and lastly the CA316 membrane (13.39%). The highest fouling index reported for the NF90 membrane could be explained due to the adsorption of organic compounds on the membrane surface through a possible formation of hydrogen bonds between the membrane polymer (polyamide) and organic compounds. Furthermore, according to the results reported by Arsuaga et al. [45], the adsorption of phenolic compounds on the membrane could be promoted by the hydrophobic interactions with the membrane material, playing an important role in the retention of solutes, a higher flux decline and a high fouling index. On the other hand, the permeate fluxes of UF and NF membranes might be severely decreased when treating low molecular weight hydrophobic solutes. In addition, the extent of the permeate fluxes reduction was affected by the hydrophilic/hydrophobic properties of the membrane material and the concentration of the solutes [46]. Accordingly, the three laboratory-made cellulose acetate membranes showed a low fouling index, when compared with the NF90 membrane.

A high recovery of the hydraulic permeability (89.41%) was observed for the CA316 membrane after cleaning with distilled water. Similar results were noticed for the CA316-70 and the CA400-22 membranes, with recoveries of 80.10% and 80.57%, respectively. According to the highest fouling index measured for the NF90 membrane, the cleaning efficiency for this membrane was the lowest, with a recovery of the initial water permeability being 60.26%. Overall, an incomplete recovery of the hydraulic permeability in the membranes could be attributed to an irreversible fouling, which is a phenomenon associated with phenolic components being absorbed on the surface of the membranes [47].

### 3.5. Analyses of Membrane Selectivity

The physico-chemical parameters of NF permeate samples in comparison to the red grape pomace extract are reported in Table 7. Minimal changes in pH values were noticed in all permeate fractions, when compared to the red grape pomace extract. Although the acids of the grape (malic and tartaric acid) were associated with a pH generally in the range of 3.2 and 4.0 [48], the pH of the present extract was due to the preparation of the buffer solution with succinic acid (C_4_H_6_O_4_) and sodium borate (Na_2_B_4_O_7_·10H_2_O), establishing the pH value of 4.0.

All membranes used allowed a significant reduction in turbidity of around 95%. In particular, the NF90 membrane allowed a reduction in turbidity of 99.12%. In parallel, the NF90 membrane showed the highest rejection coefficient towards the total polyphenols and antioxidant capacity. The rejection for total polyphenols was 97%; for the antioxidant capacity the rejections were of 74% and 100% in relation to the analysis of DPPH and ABTS, respectively. Similar results were obtained by Cassano et al. [26] in the treatment of olive mill wastewaters with the same membrane obtaining a rejection for total polyphenols of 93%. Rejection coefficients for polyphenols higher than 92% were also measured by Giacobbo et al. [49] in the treatment of winery wastewater generated in the second racking from red wine production, with a polypiperazine membrane having a MWCO of 300 Da (NF270, from DOW-Filmtec, Edina, MN, USA).

**Figure 3:**
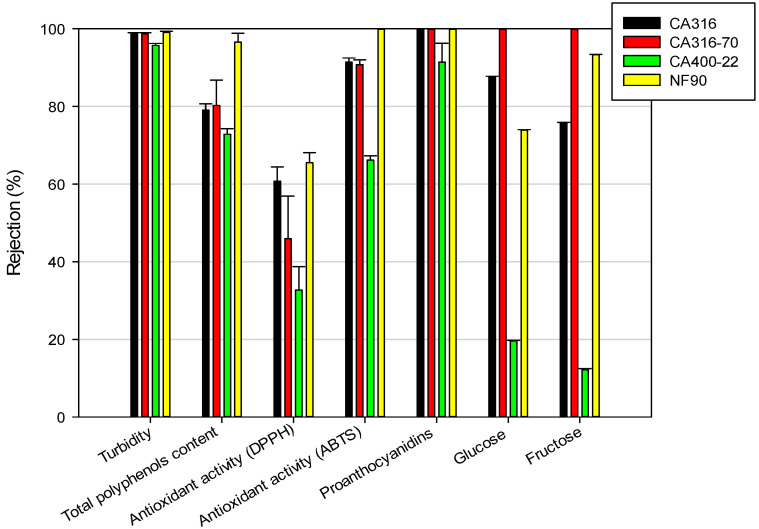
NF rejections to specific compounds and characteristics of grape pomace extract. Membrane: CA316, CA316-70, CA400-22, and NF90.

Figure 3 displays the NF membranes’ rejections to the different compounds of the grape pomace extracts. The CA400-22 membrane showed a rejection of about 73% to the total polyphenols and a rejection of about 60–70% to the antioxidant capacity. This membrane showed almost a total retention towards proantocyanidins (about 93%) and allowed to recover most parts of the glucose and fructose in the permeate stream (rejection values of 19.5% and 12.5%, respectively), obtaining a permeate rich in sugars. Therefore, this membrane offered the best performance in terms of separation between sugars and phenolic compounds. On the other hand, the CA316 and CA316-70 membranes presented a similar retention for the total phenolics (higher than 80%) and antioxidant capacity (higher than 90% in the ABTS test), as well as high retention towards glucose and fructose, with values of 87% and 100%, respectively, for the CA316 membrane, and a total retention of both compounds for the CA316-70 membrane. Similar retention values towards sugars and phenolic compounds were also measured by Galanakis et al. [50] in the treatment of winery sludge, with a composite fluoropolymer NF membrane of 1 kDa (Etna 01PP from Alfa Laval, Nakskov, Denmark). This membrane successfully separated hydroxycinnamic acid derivatives from anthocyanins and flavonols in diluted and concentrated extracts, respectively. Recently, Yammine et al. [51] evaluated the rejection coefficients of several NF membranes, with MWCO in the range of 150–1000 Da, for several families of polyphenols from grape pomace. In agreement with our findings, membranes with MWCO between 500 and 1000 Da were able to recover polymeric proanthocyanidins in the retentate stream. On the other hand, membranes with MWCO between 300 and 600 Da was useful for the fractionation of monomeric phenolic families.

## 4. Conclusions

This work proposes a sustainable valorization of grape pomace through a combination of ultrasound-assisted enzymatic extraction and membrane processing. Cellulose acetate membranes in flat-sheet configuration were prepared, characterized, and evaluated for their selectivity towards phenolic compounds, proanthocyanidins, and sugars. Their performance was compared with that of a commercial nanofiltration membrane.

All tested membranes presented high retention coefficients towards phenolic compounds and proanthocyanidins and were suitable for concentration purposes. Among these membranes, the CA400-22 membrane exhibited low retention values for glucose and fructose (rejection values of 19.5% and 12.5%, respectively) and rejections for phenolic compounds and proanthocyanidins of 73% and 92%, respectively. Therefore, this membrane is a suitable candidate for phenolic compounds/sugars fractionation. In addition, its productivity was the highest in the selected operating conditions of extract processing through nanofiltration. The overall results indicate that the combination of an ultrasound-assisted enzymatic extraction with the development of tailor-made cellulose acetate membranes could be a useful and sustainable approach for the recovery of valuable fractions from red grape pomace of interest, for the production of innovative formulations with specific requirements for the pharmaceutical and food industry.

## Figures and Tables

**Table 1:** Film casting conditions and casting solutions used for the production of nanofiltration (NF) membranes.

	Membrane Type		
	CA316	CA316-70	CA400-22
Casting solution (wt.%)			
Cellulose acetate 398	17	17	17
Acetone	69.2	69.2	61
Formamide			22
Magnesium perchlorate	1.45	1.45	
Water	12.35	12.35	
Casting conditions			
Temperature of coagulation bath solution (°C)	0	0	0
Temperature of atmosphere (°C)	20–25	20–25	20–25
Solvent evaporation time (min)	1	0.5	0.5
Gelation medium	ice cold water (1–2 h)	ice cold water(1–2 h)	ice cold water(1–2 h)
Annealing conditions			
Annealing medium		hot water	
Annealing time (min)		11	
Annealing temperature (°C)		70	

**Table 2:** Characteristics of the NF90 membrane.

Membrane Material	Aromatic Polyamide
MWCO (Da)	200 ^c^
Stabilized salt rejection (%)	>97.0 ^a^
Max. inlet pressure (bar)	41
Max. operating temperature (°C)	45
pH operating range	2–11 ^c^
Contact angle (°)	28.6 ^b^
Zeta potential at pH 7 (mV)	−9.1 ^b^

^a^ Test conditions: 2000 ppm MgSO_4_, 25 °C and 15% recovery at 4.8 bar; ^b^ Data from Kong et al. [33]; ^c^ Data from manufacturer.

**Table 3:** Rejections of NF membranes to specific solutes, salts, and ethanol (Test conditions: feed solute concentration, 600 ppm; temperature, 25 ± 1 °C; feed flowrate, 0.8 L/min; pressure, 20 bar).

Membrane Type	Saccharose (%)	Glucose (%)	Raffinose (%)	PEG (%)	NaCl (%)	Na_2_SO_4_ (%)	Ethanol (%)
NF90	100	100	100	99	95	99	50
CA316-70	98	95	98	89	77	97	7
CA316	70	50	77	55	27	86	1
CA400-22	16	11	21	20	10	47	2

PEG—polyethylene glycol.

**Table 4:** Physico-chemical characteristics of red grape pomace extract.

Parameter	Value
Turbidity (NTU)	169 ± 0.5
pH	4.0 ± 0.1
Total polyphenolics FC (mg GAE/100 g dw)	260 ± 10.3
AC DPPH (% scavenging)	16 ± 0.3
AC ABTS (% scavenging)	41 ± 3.5
Proanthocyanidins (mg CE/100g dw)	49 ± 6.2
Glucose (mg/100 g)	46 ± 0.0
Fructose (mg/100 g)	403 ± 0.1

FC: Folin-Ciocalteu; GAE: gallic acid equivalents; CE: (+)-catechin equivalents; dw: dry weight pomaces; and AC: antioxidant capacity.

**Table 5:** Average permeate flux for selected membranes in the nanofiltration of red grape pomace extract (operating conditions: pressure, 20 bar; temperature, 25 ± 1 °C).

Membrane Type	*J_p_* (L/m^2^ h)
NF90	26.09 ± 1.25 ^C^
CA316-70	43.38 ± 0.9 ^B^
CA316	44.44 ± 1.05 ^B^
CA400-22	50.58 ± 2.55 ^A^

Different superscript letter in properties are significantly different according to the Tukey’s HSD test (*p* ≤ 0.05).

**Table 6:** Hydraulic permeabilities in NF/cleaning cycles, fouling index, and cleaning efficiency of the selected membranes.

	Membrane Type			
	CA316	CA316-70	CA400-22	NF90
*W_p_*_0_ (L/m^2^ h bar)	4.63	4.42	8.34	3.75
*W_p_*_1_ (L/m^2^ h bar)	4.01	2.83	6.39	2.23
*W_p_*_2_ (L/m^2^ h bar)	4.14	3.54	6.72	2.26
Fouling index (%)	13.39	35.97	23.38	40.53
Cleaning efficiency (%)	89.41	80.10	80.57	60.26

*W_p_*_0_, water permeability before the NF of grape pomace extract; *W_p_*_1_, water permeability after the NF of grape pomace extract; and *W_p_*_2_, water permeability after cleaning with distilled water.

**Table 7:** Physico-chemical characteristics of feed and permeate samples obtained in the NF of grape pomace extract.

Parameter	Feed	Permeate			
		CA316	CA316-70	CA400-22	NF90
Turbidity (NTU)	169 ± 0.5 ^A^	2.9 ± 0.4 ^C^	2.2 ± 0.5 ^C^	7.2 ± 0.9 ^B^	1.5 ± 0.4 ^C^
pH	4.0 ± 0.1 ^A^	3.7 ± 0.1 ^AB^	3.7 ± 0.2 ^B^	3.8 ± 0.1 ^AB^	3.6 ± 0.0 ^B^
Total polyphenol FC (mg GAE/100 g)	260 ± 10.3 ^A^	54 ± 4.1 ^B^	50 ± 15.1 ^B^	70 ± 2.7 ^B^	9.1 ± 6.3 ^C^
AC DPPH (% scavenging)	16 ± 0.3 ^A^	6.4 ± 0.4 ^CD^	8.9 ± 2 ^BC^	11 ± 0.9 ^B^	5.6 ± 0.3 ^D^
AC ABTS (% scavenging)	41 ± 3.5 ^A^	3.5 ± 0.1 ^C^	3.8 ± 0.8 ^C^	13.9 ± 0.7 ^B^	n.d
Proanthocyanidins (mg CE/100 g dw)	49 ± 0.7 ^A^	n.d	n.d	4.0 ± 1.7 ^B^	n.d
Glucose (mg/100 g)	46 ± 0.0 ^A^	5.7 ± 0.0 ^D^	n.d	37 ± 0.0 ^B^	12 ± 0.0 ^C^
Fructose (mg/100 g)	403 ± 0.1 ^A^	98 ± 0.2 ^C^	n.d	354 ± 0.4 ^B^	27 ± 0.0 ^D^

Different superscript letter in properties are significantly different according to the Tukey’s HSD test (*p* ≤ 0.05). FC, Folin-Ciocalteu; GAE, gallic acid equivalents; CE, (+)-catechin equivalents; dw, dry weight pomaces; and AC, antioxidant capacity.

## Supplementary Materials

The following are available online at https://www.mdpi.com/2304-8158/9/11/1649/s1. Figure S1: Pure water permeability of the NF90 membrane. *W_p_*_0_, water permeability before the red grape pomace extract nanofiltration; *W_p_*_1_, water permeability after the red grape pomace extract; and *W_p_*_2_, water permeability after cleaning with water (operating conditions—feed flowrate of 0.8 L/min; Tof 25 ± 1 °C); Figure S2: Pure water permeability of the CA316 membrane. *W_p_*_0_, water permeability before the treatment of red grape pomace extract; *W_p_*_1_, water permeability after the treatment of red grape pomace extract; and *W_p_*_2_, water permeability after cleaning with water (operating conditions—feed flowrate, 0.8 L/min; T, 25 ± 1 °C); Figure S3: Pure water permeability of the CA316-70 membrane. *W_p_*_0_, water permeability before the treatment of red grape pomace extract; *W_p_*_1_, water permeability after the treatment of red grape pomace extract; and *W_p_*_2_, water permeability after cleaning with water (operating conditions—feed flowrate, 0.8 L/min; T, 25 ± 1 °C); and Figure S4: Pure water permeability of the CA400-22 membrane. *W_p_*_0_, water permeability before the treatment of red grape pomace extract; *W_p_*_1_, water permeability after the treatment of red grape pomace extract; and *W_p_*_2_, water permeability after cleaning with water (operating conditions—feed flowrate, 0.8 L/min; T, 25 ± 1 °C).
