# Supplementary material for: Recovery of Phenolic Compounds from Red Grape Pomace Extract through Nanofiltration Membranes
Source: Foods. 2020 Nov 12;9(11):1649. doi: 10.3390/foods9111649 (PMC7697400; doi:10.3390/foods9111649)
Supplement: Supplementary file 1 [file foods-09-01649-s001.pdf]

foods MDPI

SUPPLEMENTARY DATA

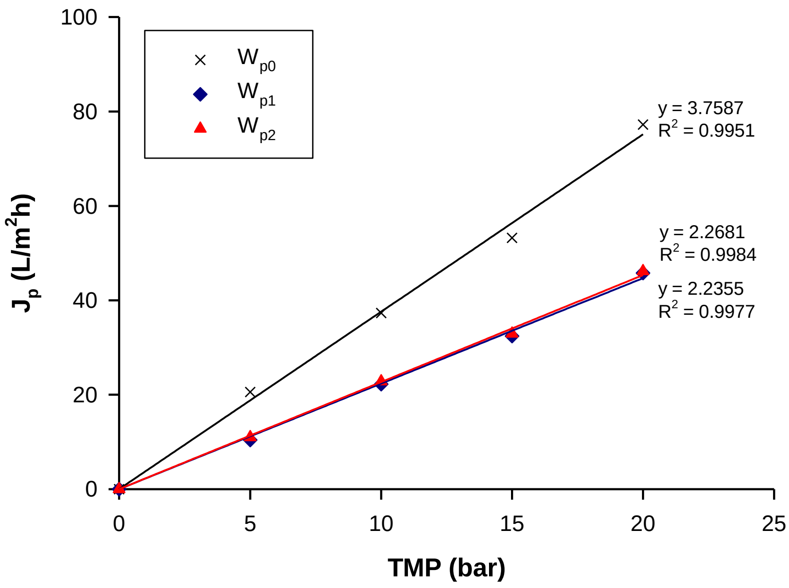

Figure S1. Pure water permeability of the NF90 membrane. W_{p0}, water permeability before the red grape pomace extract nanofiltration; W_{p1}, water permeability after the red grape pomace extract; W_{p2}, water permeability after cleaning with water (Operating conditions: feed flowrate, 0.8 L/min; T, 25±1 °C).

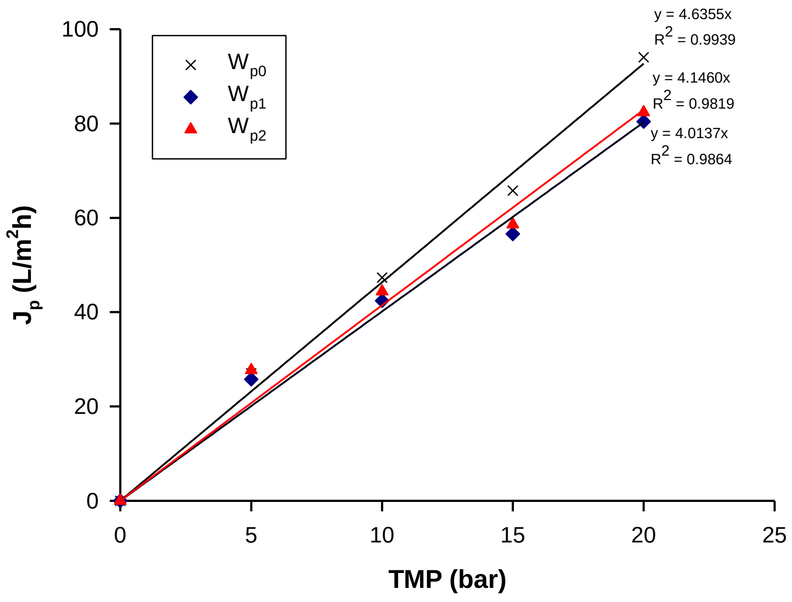

Figure S2. Pure water permeability of the CA316 membrane. W_{p0}, water permeability before the red grape pomace extract nanofiltration; W_{p1}, water permeability after the red grape pomace extract; W_{p2}, water permeability after cleaning with water (Operating conditions: feed flowrate, 0.8 L/min; T, 25±1 °C).

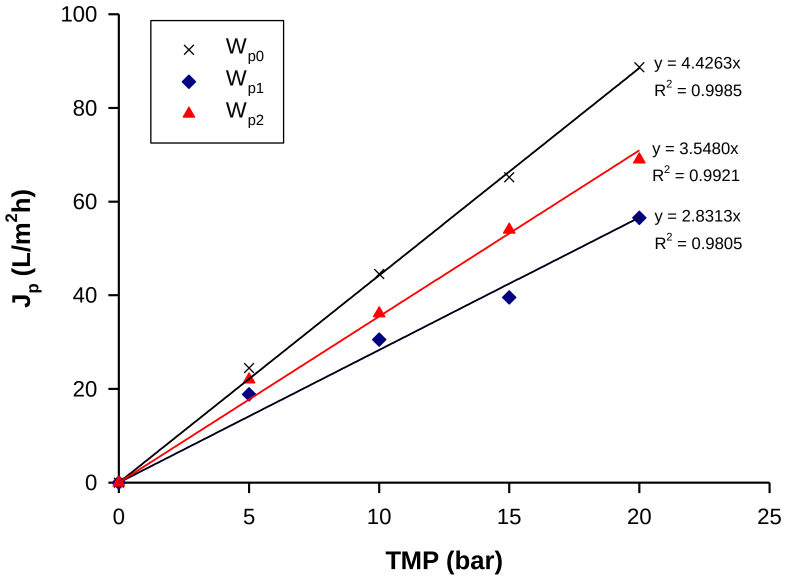

Figure S3. Pure water permeability of the CA316-70 membrane. W_{p0}, water permeability before the red grape pomace extract nanofiltration; W_{p1}, water permeability after the red grape pomace extract; W_{p2}, water permeability after cleaning with water (Operating conditions: feed flowrate, 0.8 L/min; T, 25±1 °C).

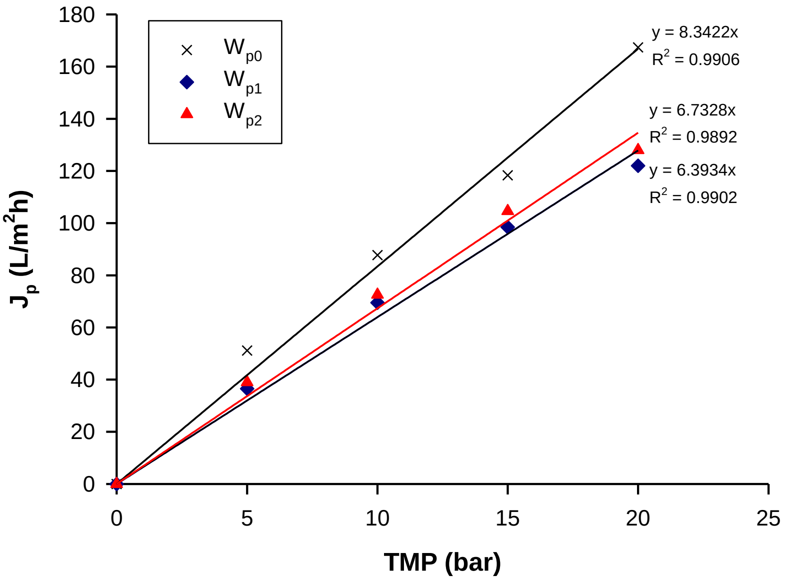

Figure S4. Pure water permeability of the CA400-22 membrane. W_{p0}, water permeability before the red grape pomace extract nanofiltration; W_{p1}, water permeability after the red grape pomace extract; W_{p2}, water permeability after cleaning with water (Operating conditions: feed flowrate, 0.8 L/min; T, 25±1 °C).
